# Exploring Farmers’ Reasons for Antibiotic Use and Misuse in Pig Farms in Brazil

**DOI:** 10.3390/antibiotics10030331

**Published:** 2021-03-22

**Authors:** Rita Albernaz-Gonçalves, Gabriela Olmos, Maria José Hötzel

**Affiliations:** 1 Instituto Federal Catarinense, Campus Santa Rosa do Sul, Santa Rosa do Sul, SC 88965-000, Brazil; rita.silva@ifc.edu.br; 2 Laboratório de Etologia Aplicada e Bem-Estar Animal, Departamento de Zootecnia e Desenvolvimento Rural, Universidade Federal de Santa Catarina, Florianópolis, SC 88034-001, Brazil; 3 Veterinary Epidemiology Unit, Department of Clinical Sciences, Swedish University of Agricultural Sciences, Box 7054, 75007 Uppsala, Sweden; gabriela.olmos.antillon@slu.se

**Keywords:** antimicrobial, AMU, AMR, attitudes, livestock, prudent use of antibiotics, swine

## Abstract

Stressful management that makes farmed pigs susceptible to infections is associated with high antibiotic use (AMU) and resistance (AMR). Pig farmers are key stakeholders to support the international agenda pushing AMU restrictions. We interviewed 58 pig farmers on AMU/AMR, biosecurity, veterinary assistance, disease prevention and treatment, aiming to understand practices and attitudes towards the AMU/AMR problem. Farmers described a reliance on antibiotics to prevent and treat disease while neglecting biosecurity measures. We identified inappropriate AMU practices (high use of broad-spectrum antibiotics, incorrect dosage or treatment length) and unrestricted access to antibiotics, which encouraged imprudent AMU. Nevertheless, most farmers considered this AMU legitimate to guarantee herd productivity and showed unpreparedness and resistance to changing AMU practices, perceiving limitations (economic, sanitary and inspection) more easily than alternatives to reduce AMU. Agro-industries and foreign markets were mentioned, and internal consumers dismissed as potential motivators for behavioral changes. Importantly, farmers’ economic, technical and social factors may limit their autonomy to change practices. We conclude that the observed distancing of pig farmers from the AMU/AMR problem limits the efficiency of policies aiming for a prudent AMU. Our study indicates a need for education, training and behavior change nudging that should include other stakeholders beyond farmers.

## 1. Introduction

The definition of antimicrobial resistance (AMR) proposed by the Global Action Plan [1] refers to the resistance acquired by several microorganisms (bacteria, fungi, viruses and parasites) to antimicrobials. This concept covers a range of drugs; however, the focus of the present study is on antibiotics, due to the high level of use and importance that these drugs have in the swine production chain [2,3]. The antibiotic use (AMU)/AMR problem is often considered a health problem; yet, building evidence indicates that AMR is an evolutionary challenge accelerated by social, cultural and economic factors that lead to the misuse, overuse and abuse of these life-saving medicines [4]. Scientific evidence links the use of antibiotics in livestock with the risk of transmission of antibiotic-resistant bacteria to humans [5,6] mainly due to the use of antibiotics in low doses in the diet of animals [5,7]. AMR transmission can occur through direct contact with contaminated people, animals and food, or through the environment, via animal waste containing resistant bacteria that may contaminate soil and water [3,6,8]. Antibiotic residues can also induce the selection of resistant bacteria in the environment. For instance, the presence of antibiotic residues and genes of resistant bacteria has been detected in surface waters supplying urban areas in China and the Netherlands [9,10]. Irrigation water and manure used for fertilization may also contain residues of antibiotics that will be absorbed by the soil and plants [11]. This complex and intricate relationship between human behavior and human, animal and environmental health strengthens the need for multidisciplinary approaches to tackle AMR.

Although there is an international mobilization to encourage measures of prudent use of antibiotics in livestock, in low- and middle-income countries sales of veterinary antibiotics are unregulated [12]. Several studies in these countries show a relationship of antibiotic dependence, mainly in pig farming [13,14]. The WHO, FAO and OIE consider AMR a global health emergency, which has led to the development of policies aiming to reduce the use of antibiotics in several countries [1,15,16]. This stance can force countries that rely on export livestock products to conform to international recommendations for prudent use of antibiotics. Brazil is the fourth largest producer of pork in the world and a signatory to the OIE, and its economy is highly dependent on the export of its agricultural products [17]. The PAN-BR is a plan put forward by the Brazilian Health Ministry together with other government entities to adapt to the practices of rational use of antibiotics in the coming years [18]. Pressure from foreign markets may require Brazilian health agencies and the animal production chain to rapidly adapt to the international scenario of restriction of the use of antibiotics [19]. These changes require rethinking the current production systems that rely on high antibiotic use. 

Many Brazilian pig farmers are subordinated to industrial groups that establish rules for pig rearing. These farmers, as well as independent pig farmers, will be directly responsible for implementing measures to cope with the restriction of the use of antibiotics. Thus, it is important to understand the knowledge and opinions of this group, as they are mediators and guardians of animal health, and likely the most affected by the burden of change. Qualitative social research brings a different perspective to the use of antibiotics that can help us to understand the attitudes of farmers in relation to this topic, especially about how they perceive antibiotics in their routine and their expectations in relation to a scenario of change [20,21]. There is a need to understand how Brazilian pig farmers feel about this problem, if they see the need to change and, if so, whether they are prepared to modify their practices in order to reduce the use of antibiotics. Thus, the aim of this study was to explore the knowledge, attitudes and practices of pig farmers regarding the use of antibiotics in pig farming, as well as regarding bacterial resistance to antibiotics.

## 2. Materials and Methods

This study, conducted by the Applied Ethology Laboratory of the Federal University of Santa Catarina (LETA-UFSC), is part of the research project entitled “Knowledge and attitudes of Santa Catarina’s pig industry on antibiotics, bacterial resistance and animal welfare”. Thus, some details of the methodology are similar to those presented Albernaz-Gonçalves et al. [22]. This particular study followed a qualitative approach, using in-depth semi-structured interviews to understand pig farmers’ knowledge and attitudes about antimicrobial resistance (AMR) and prudent use of antibiotics.

### 2.1. Study Location

Pig farming is one of the main Brazilian livestock and agribusiness activities. Brazil holds the fourth position as a global pork producer and exporter [23]. Santa Catarina (SC) is the Brazilian state with the largest production in Brazil, housing 25% of the sows [24]. Braço do Norte, located between 28°16’30” S and 49°09’56” W, is a municipality belonging to the micro-region Tubarão, which is the second-largest pork-producing region in Santa Catarina. Tubarão has 19 municipalities and around 1500 registered pig production units, housing a total of 100,000 sows. This site was chosen for the study because it presents intensive pig farming systems that are relatively diverse regarding labor type, herd size, production types and production models (Table 1). Pig production in the southern region of Brazil is characterized by specialized production segregated into several breeding sites and some full-cycle farms. Most (~70%) of the farms in the studied region are considered medium-sized, i.e., house between 300 and 1000 housed pigs; the predominant production models are integrated pig farmers (62%), cooperatives (26%) and independent producers (12%) [24]. Braço do Norte specializes in the production of weaned piglets for fattening, but it also has full-cycle farms that sell finished pigs for slaughter in small local slaughterhouses.

### 2.2. Participants’ Recruitment

The interviews were done face-to-face between January and February 2019; all interviews were carried out in the Brazilian Portuguese language by the same person (R.A.-G). Before beginning the interview, the participant was given and read a free informed consent form, which contained all the information relevant to the interview. The audio recording and interview process started only after the interviewee(s) understood and signed the consent form. Participants were invited to ask questions, interrupt the interview or withdraw from the study at any time. The average duration of the interviews was 34 min per interview (between 12 min and 1 h 20 min).

The first participants were recruited through a network of contacts of the first author (former students resident in the region). These informants indicated potentially interested farmers, of which 12 accepted to participate. Other farmers were identified using a non-probabilistic snowball sampling method, which is a method indicated for accessing information from difficult-to-reach groups [25]. Difficulties in accessing farmers included an outdated list of breeders’ associations, distance from urban centers and lack of Internet and telephone access. Initial contact with producers was done over the phone or on a first a visit to the farm to make the invitation and arrange the interview.

From a total of 63 visits, 58 interviews were completed. The interviews were conducted in two stages: first, we interviewed 40 farmers, analyzed the responses obtained and returned to the study region to carry out more interviews; after the second analysis of another 23 interviews, as we did not identify new elements in the participants’ responses, we considered that the number of interviews ensured good data saturation and an in-depth, diverse and rich report on the topic. The sample size for qualitative research depends on the diversity of the population studied and the amount and wealth of data collected from each participant [26,27]. The participants in this study provided a rich sample of data as they represented a plurality of production contexts and demographic data (Table 1 and Table 2).

### 2.3. Interview Script

The interview script contained semi-structured and open-ended questions (Tables S1–S3). The interview was divided into five sections, which corresponded to (1) socio-demographic issues, (2) biosecurity practices, (3) forms of disease prevention and control, (4) means of diagnosis, treatment and technical assistance and (5) knowledge and attitudes about bacterial resistance to antibiotics. At the end of the interviews, participants were presented with a hypothetical scenario of prudent use of antibiotics and were asked two questions: “If in the future the use of antibiotics as growth promoters and prophylactics were not allowed, and the use of parenteral antibiotics controlled, what would be the impacts of this scenario for pig farming in Brazil?; What measures would be necessary to reduce the use of antibiotics in pig farming?”.

### 2.4. Data Analysis

To analyze the material obtained in the interviews, we used an inductive (reflexive) thematic analysis approach, following the analysis proposed by Braun and Clarke [26] and Braun et al. [27]. This type of investigation is not associated with any specific theoretical framework and provides a flexible and varied approach beyond the researcher’s insights or expectations. In order to define and code the themes of the inductive thematic analysis, the authors made an exhaustive reading of the transcripts to become familiarized with the data. In the inductive approach, the analytical process starts from the data, working “bottom-up” and, therefore, is based on the responses of the interviewees to minimize bias. Each theme was refined through interactive discussion between the three authors, and names (titles) and clear definitions for each theme were created. The analysis was carried out with the aid of the NVivo qualitative data management program (version 11, 2015; QSR International Pty Ltd., Doncaster, VIC, Australia). The selected excerpts representing the themes were translated into English by MJH and revised by GO. 

Quotes are displayed in Appendix A, and are cited in the Results section by farmer number and order of appearance (e.g., F30a refers to the first excerpt from the interview with Farmer 30; F3b is the second excerpt quoted from the interview with Farmer 3). 

## 3. Results

### 3.1. Demographic Data and Characterization of Farms

Demographic and farm data are shown in Table 1 and Table 2. The visited farms had between 50 and 1200 sows or finishing pigs, including full-cycle (or farrow-to-finish) farms, piglet-producing units (or breeding farms), growers and fattening units. In general, the interviewees considered themselves as experienced pig producers, with 82% of the participants stating having more than 10 years of experience in the industry.

Some of the farmers complemented their income from pig farming with other work/activities, with dairy cattle present in most farms. Family members performed the farm labor but some also hired staff to aid on the farm. In most of the visited farms, a man was responsible for running the farm, although in these farms women were also included in the routine of the farms, mainly in the care of dairy cattle.

### 3.2. Pig Production Models and Purchase of Antibiotics

The farmers that participated in this study belonged to three distinct production models: some worked independently (*n* = 36) and some were associated with one of the three integrators (*n* = 15), or one of the two cooperatives (*n* = 7) involved in pig production in the area. Integrated and cooperated farmers met criteria established by the Ministry of Agriculture, Livestock and Supply (MAPA) to sell their products nationally and internationally. Independent farmers worked with the municipal or state inspected slaughterhouses, which supplied the Brazilian domestic market. In the piglet-producing units, the piglets were weaned and transported to fattening farms located in western Santa Catarina.

**Figure 1:**
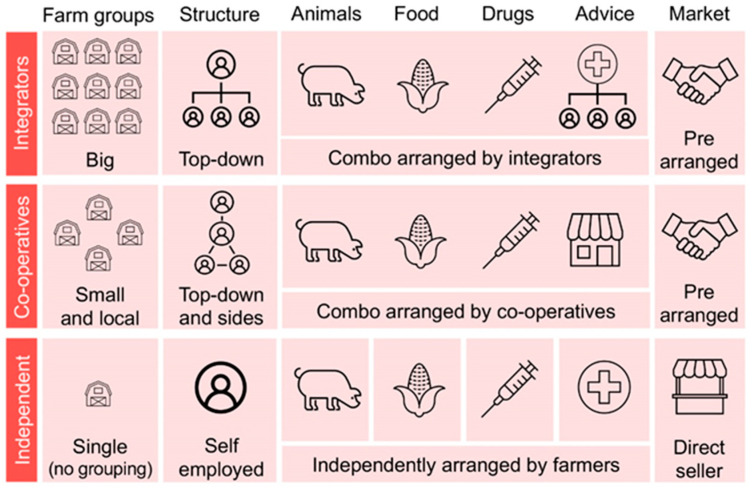
Schematic representation of pig production models in Brazil.

Many practices were shared across farmers regardless of the production model; however, the way farmers acquired inputs and sold their products differed according to the production model (see Figure 1). In summary, all farmers linked to integrators had a pre-agreement with agro-industries whereby the company provided basic inputs (pigs, feed, medicines) and technical assistance, while the farmer was responsible for the infrastructure, labor and supply of animals for slaughter. Integrators are big companies that have farmers at their command; on the other hand, cooperatives are organizations formed by groups of associated farmers, which work in a similar way to the integration model but at a local level. Independent farmers had no contractual ties to specific agro-industries or cooperatives, and therefore were not subject to the same work and organization rules as the other groups, and were responsible for purchasing inputs and selling their animals (Figure 1).

Most of the independent farmers (67%) produced their animal feed on their farms from grains they produced themselves and some additional external inputs (e.g., vitamin and mineral ingredients) purchased from local shops or through vendors. When they considered it necessary to add antibiotics to the feed for group treatment or for prophylactic use, independent farmers bought the powdered antibiotics for mixing in feed. It is important to note that these farmers were not complying with the norms of IN65/2012/MAPA (F30a). However, their reports indicated that they were not sufficiently informed about these regulations and that they did not consider this practice incorrect. The supply of antibiotics for feed by shops or through vendors also breached the rules and indicates a lack of inspection by health agencies in commercial establishments. Independent farmers also bought injectable antibiotics from local agricultural shops (53%) or through vendors of nutritional inputs (45%). Farmers who received vendors on their farm informed us that they placed orders via cell phone messages. According to these farmers, there was no difficulty in acquiring antibiotics (F57a) and no participant reported being required to have a veterinary prescription for the purchase of medications at shops or from vendors.

The integrated farmers received from the company, together with the feed, a package with all veterinary antibiotics and a list of medications recommended for each situation. In this list, signed and stamped by a responsible veterinarian, there was a list of clinical signs, respective probable diseases and recommended medications (antibiotics, anticoccidials, anti-inflammatories and disinfectants), with the concentrations of each active ingredient and recommended dose. As a company rule, farmers from integrators could not purchase veterinary medicines on their own. The companies informed their members when antibiotics were included in their feed, according to the recommendations of field technicians and veterinarians (F50a). Farmers from cooperatives also received lists of medications, or received medications and feed, medicated or not, from the cooperative; however, they were also allowed to purchase these inputs at shops.

### 3.3. Level of Adoption of Biosecurity Practices on Farms

Biosecurity practices adopted on the farms, as described by the farmers, are shown in Table 3. Following biosecurity protocols, the presence of the research team during the visits was restricted to the external environments of the farms. Although unauthorized persons were not allowed to enter the farms, we did not observe any physical barriers (bars, screens, green barriers) or wheel dip. The information we obtained about vaccination was inaccurate, partly because of the confusion that many farmers had between injectable antibiotics and vaccines (F3a), so we did not consider data on that topic.

Few farmers cited basic biosecurity practices such as treatment of drinking water with chlorine, or control of rats, visitors and vehicles (F1a; F2a). Few farms kept the farm facilities empty for longer than 7 days after cleaning and disinfection ahead of initiating a new productive cycle. Furthermore, none had quarantine protocols for the introduction of new animals to the herd. Replacement gilts were housed in pens separated from the older sows, but shared the same environment with sows and breeding males.

### 3.4. “Antibiotic Shocks” and the Trivialization of Antibiotic Therapy

Farmers used antibiotics for therapeutic and prophylactic treatments and as growth-promoting additives. All farmers mentioned the use of antibiotics as the main way to prevent infections in pigs and considered antibiotics indispensable in pig farming (F3b; F19a). Only 19% of the farmers acknowledged using antibiotics as growth promoters when they were not using them for prophylactic treatments, and 29% did not know if their diets contained growth promoters.

In the farrow-to-finish and breeding farms, 85% of farmers used what they called “antibiotic shocks” to prevent genitourinary infections in the sows (Table 4). They alternated different antibiotics for the sows in each cycle of medication (F2b; F57b). Farmers rarely mentioned preventive measures for breeding sows that did not involve antibiotics; for example, only 7% mentioned vaccinations and 5% used organic acids and prebiotics. Sows that showed evident symptoms of untreatable/uncontrollable infections were culled (F15a).

Weaned piglets also received prophylactic antibiotics continuously in the diet or as “antibiotic shocks” (Table 4). All farmers described alternating antibiotic groups, that is, with each feed batch, the piglets received a different antibiotic. For this reason, a piglet could have contact with six or more antibiotic groups between 28 and 70 days of life (F17a; F19b). The groups of antibiotics most cited were aminopenicillins, tetracyclines and amphenicols for use in diets; quinolones, aminopecillins and macrolides for injectable use (Table 5). Farmers used injectable antibiotics to prevent infections in newborn piglets, before weaning the piglets and when replacement gilts were moved to the sows’ building (F41a; F60a; F3c; F18a; F35a).

Some farmers claimed to control piglet diarrhea with antibiotics in the feed of lactating sows (F61a). Farmers also used antibiotics to treat individual cases of neonatal pneumonia and diarrhea. When more than one pig in the group had symptoms, the entire group was given therapeutic doses of antibiotics via feed or water. 

Some statements suggest that the pigs were exposed for long periods to large amounts of antibiotics (F18b). Farmers expressed a social conformity in the use of antibiotics as a preventive strategy. Yet, the ways of conveying their behavior indicate that they downplayed an action that they found unusual themselves (F30b). This was also identified in the constant use of the term “antibiotic shocks” to refer to the strategic use of antibiotics in pig diets for preventive purposes, and reference to antibiotic therapy as a simple and routine practice (F51a; F46a).

Although 72% considered the cost of antibiotics to be high (F18c; F35b), farmers said that spending money on antibiotics was necessary to avoid the risk of losses due to disease or increased mortality (F42a; F31a). In other words, in the view of these farmers, antibiotics were a “necessary evil”.

### 3.5. Disease Diagnosis, Drug Prescription and Farmer/Veterinarian Assistance

All farmers received some type of veterinary technical assistance, through nutrition input companies, integrators or cooperatives (Table 6). Some nutrition or pharmaceutical companies provided free assistance to farmers who purchased their products. According to the farmers, in most cases, field technicians or veterinarians visited the farms weekly or only in emergencies. Integrated farmers linked to cooperatives had periodic visits, while independent farmers relied on visits by vendors or called veterinarians, which for some limited access to qualified information (F57c).

Most farmers claimed to be able to identify diseases and choose treatments according to their own experience, as show in Table 7. They decided on the doses of antibiotics following recommendations from the label or in the lists provided by the companies. We observed some flaws in the use of antibiotics, such as the choice of inappropriate active ingredients, incorrect use of doses and insufficient treatment time; farmers also used antibiotics to treat viral infections, indicating that they had difficulty in distinguishing bacterial from viral infections (F61b; F41b; F60b; F35c; F18d). Few farmers mentioned using antibiotic sensitivity tests, and this only happened in specific cases when farmers were facing health challenges that were difficult to control on their farms (F59a).

### 3.6. AMR Expressed as A Failure of the Antibiotics, Not Human Actions

Forty-five percent of farmers did not know if there were similarities between antibiotics for human and veterinary use, while 26% believed there were some similarities, and 21% said they were the same drugs (F35d). Some of them believed that even if antibiotics were shared between species there would be no risk to consumers, as long as the withdrawal period before slaughter was respected. Farmers defined AMR as the failure of antibiotics to control disease (F1a; F14a). As contributing factors to the development of AMR, farmers mentioned the continued use of the same active ingredients (74%), incorrect dosage (14%), unnecessary use of antibiotics (5%) and incorrect treatment time (5%). Some farmers associated AMR with low immunity of pigs (F58a; F37a).

### 3.7. Farmers’ Perceptions of Consumers’ Beliefs Regarding AMU

About half of the interviewed farmers (53%) believed that consumers were unaware or unconcerned about the use of antibiotics in pigs. In their opinion, consumers were oblivious and disconnected from the rural reality and were concerned mostly with the price and quality of the goods. Yet, a third (36%) of the farmers mentioned that they believed that consumers cared about how antibiotics were used on their farms and linked these beliefs with concerns of maintaining a positive image of pork among consumers and with the potential traceability of problems that could be associated with their farms (F54a; F20a). 

Moreover, some farmers told us that they did not eat pork from animals that received antibiotics, and some of them stated that they fattened animals without antibiotics for their own consumption (F25a; F23a; F61c).

### 3.8. Reducing AMU—A Distant Idea for Farmers

Farmers expressed divided opinions about the prudent use of antibiotics in Brazilian pig farms. For 48% of farmers, the use of antibiotics in pig farming in Brazil was adequate, and 43% believed that the use was not rational (9% did not know how to express an opinion on this subject). Sixty-six percent of farmers had some knowledge about prudent antibiotic use policies and 63% showed negative attitudes towards a hypothetical scenario of restriction of the use of veterinary antibiotics; even those favorable to these measures did not consider it a viable scenario. Farmers cited economic, productive, health and cultural barriers to adopting changes in the use of antibiotics. In their opinion, limiting the use of antibiotics for prophylactic purposes would increase production costs and undermine small farmers’ survival (F38a). They believed that removing antibiotics would aggravate the economic crisis and demand capital for structural investments (F4a); some mentioned problems with biosecurity (F2c; F1b; F58b).

In the assessment of some farmers, cultural elements such as Brazilians’ disregard for rules and the farmers’ dependence on the use of antibiotics made measures of the prudent use of antibiotics unfeasible (F9a; F32a). The difficulties of the competent bodies to inspect compliance in the national territory were also raised as an obstacle that would prevent full control of the use of antibiotics (F42b).

One view identified in the group was that the prudent use of antibiotics in animal production in Brazil depends on changes in current production models (F16a). Other suggestions for changes to reduce dependence on antibiotics were the use of pigs genetically more resistant to diseases, natural additives or improvements in animal welfare (F33a; F22a).

## 4. Discussion

The information provided by the farmers in this study suggests that their pigs were exposed to large amounts of antibiotics for long periods. When asked about the general use of antibiotics on pig farms, almost half of the farmers considered that the use was indiscriminate; however, they attributed this reckless conduct to other farmers and not to themselves. Antibiotics were part of a repertoire of routine practices, which these farmers considered legitimate and beneficial. In addition to relying on the effectiveness of antibiotics, pig farmers found it difficult to change deep-rooted habits. Changing routine behaviors is a challenge, especially when what is expected is a drastic change with results that are difficult to perceive [28,29], as is the case with AMR. The relatively low adoption of biosecurity and hygiene measures, the constant use of the term “antibiotic shocks” to refer to the strategic use of antibiotics in pig diets for preventive purposes and reference to antibiotic therapy as a simple and routine practice suggest a social conformity with the use of antibiotics as a preventive strategy. This, in turn, explains the low support expressed for policies aiming at reducing AMU. Pig farmers saw more advantages than risks in the AMU, and considered the cost of antibiotics high, however justified due to their efficiency; this illustrates the dependence on antibiotics of modern livestock production systems, in which the advantages of use are more noticeable than their harmful consequences.

Our findings that pigs received preventive doses of antibiotics for a large proportion of their lives suggest that focusing public policies of prudent AMU on reducing or banning antibiotics use for growth promotion may be insufficient and disconnected from the actual use of antibiotics on Brazilian pig farms. Instead, it is important to regulate the use of preventive and curative antibiotics on pig farms. In Brazil, there are no restrictions on the therapeutic and prophylactic use of antibiotics; Brazilian regulations on antimicrobials refer to technical standards for the manufacture of medicated feed and other regulatory instructions that limit or prohibit the use of certain active ingredients as growth-promoting additives [30]. Importantly, some of the antibiotics cited by the farmers in this study are classified by the WHO and OIE as “Highest Priority Critically Important Antimicrobials” [31,32]. Policies aiming at the prudent use of antibiotics do not recommend the use of several principles mentioned by farmers, such as aminopenicillins, tetracyclins, macrolides, quinolones and amphenicols for the prevention or treatment of pigs, given their association with AMR in humans. Other studies identified the use of the same active principles in pig farms in Brazil and in other countries [14,33,34]. The choice of broad-spectrum active ingredients may suggest the presence of AMR in these herds. Kirchhelle [7] warned of the risks of running out of broad-spectrum antibiotic options, as the ability of bacteria to become resistant is more efficient than the speed of pharmaceutical companies in developing new drugs.

Low adoption of biosecurity and hygiene measures was allied to the excessive use of antibiotics. Additionally, inappropriate practices identified include continuous use of preventive antibiotic therapy, “antibiotic shocks” (i.e., strategic periodic metaphylactic treatment), inappropriate dosages and dilutions and insufficient treatment time when using injectable antibiotic treatments. Excessive AMU in livestock production is discussed as a low-cost substitute for good practices including good hygiene measures to prevent infections in livestock [5] as confirmed in this study and in others carried out in Brazil [34]. Incentives to reduce AMU result in the adoption of more costly or laborious alternatives to control infection, such as vaccinations, reducing stocking density and cleaning [35,36]. Those studies further confirm that antibiotics are often used as substitutes for these practices and that it is possible to reduce AMU when these practices are adopted. Farmers were aware of the need to improve the biosecurity conditions on their farms; however, like French pig farmers [37], they showed negative attitudes towards adopting biosecurity measures, because they considered them burdensome and laborious. Many management practices used in the visited farms, like early weaning, repeatedly mixing unknown animals and cage housing for sows, negatively impact pig welfare and are associated with high levels of stress [38]. However, as shown in our accompanying study [22], farmers were not motivated to introduce practices aimed at improving welfare. The prophylactic use of antibiotics is still widely present in the pig production chain in several countries [33,39], even with several studies showing that good husbandry practices and biosecurity allow reduced use of antibiotics [40,41]. For example, in a comparative study among herds in European countries (Belgium, France, Germany and Sweden), late weaning and investing in efficient external biosecurity measures helped farmers reduce the use of antibiotics [42]. Other research showed that it is possible to avoid prophylactic use of antibiotics with low productive and economic impacts, provided that the management and welfare of pigs is improved [43,44]. 

Farmers felt confident and able to diagnose diseases and medicate animals without the need for a veterinarian and took on that responsibility, as identified in other studies [14,33,45,46]. Additionally, confidence in technical assistance was compromised by commercial conflicts of interest, since the sale of products was associated with veterinary assistance in most cases. This was reinforced by the practice of agro-industries and cooperatives to pass on lists of symptoms and medicines for farmers to apply on the farm. Thus, in this community, veterinarians lost the status of guardians of animal health and became sellers, similar to other studies carried out on the Belgian–Dutch border and in Thailand, Cambodia and China [13,14,33,47]. Compliance with the techniques recommended by the veterinarian is associated with the level of confidence that the farmer has in relation to the behavior and competence of the veterinarian [48]. Mistrust in the quality of technical assistance weakens bonds of trust between farmers and veterinarians, with negative consequences for the implementation of policies for the prudent use of antibiotics. 

Negative attitudes and skepticism regarding the policies to restrict AMU and prudent AMU in Brazil were not surprising, given the scenario described about farmers’ knowledge about AMU and AMR, added to the perception expressed by many that they made a rational use of antibiotics. Additionally, the farmers presented many arguments to justify their position against the policies of prudent use of antibiotics, including financial insecurity of the sector, the increase in production costs and the health problems present in the herds. Farmers in other countries have also identified the same economic and health barriers as important limitations to restricting antibiotics in pig farming [20,49]. As in the study by Golding et al. [49], farmers showed mistrust in the capacity of government agencies to inspect and enforce rules, which contributed to their negative attitudes towards policies of prudent use of antibiotics. Failure to enforce rules was revealed in the farmers’ access to antibiotics from vendors without prescription, as reported previously [50,51]. Indeed, systems of control of the prescription and sale of antibiotics are considered essential to the implementation of policies for the prudent use of antibiotics [52,53]. In contrast, free access to antibiotics without sales control encouraged the imprudent AMU by the farmers in the present study.

The lack of knowledge about various aspects of AMU and AMR may explain why, although farmers identified some triggering factors for AMR, many failed to establish a connection between AMR and the continued use of antibiotics they described in the interviews. Most farmers did not see a relationship between human and veterinary antibiotics or the AMU in livestock as a risk factor for AMR in humans. Other studies have also shown that farmers either ignored [33] or showed skepticism about the role of intensive livestock farming as a contributing factor to the spread of AMR [49,54]. Some farmers acknowledged the risk of antibiotic residues, but exclusively residues in meat associated with not meeting the antibiotic grace period, similar to other studies [55]. The farmers’ lack of knowledge about the risks of AMR put their health at risk, given the importance of occupational transmission of AMR [56,57]. In addition, the lack of awareness about AMR can hinder the implementation of practices aligned with the prudent use of antibiotics [49,58]. 

Farmers in this study seemed unwilling to make changes that are needed to allow reducing of AMU. Underlying this resistance to change was the feeling that reducing AMU under the current circumstances would be impossible. In our opinion, farmers were correct in their arguments that it is impossible to sustain the current production system without high amounts of AMU. Furthermore, we identified economic, technical and social factors that limit farmers’ autonomy and power to change practices. As discussed by others [7,59,60] antibiotic restriction policies based on individual attitudes may not be enough to solve the AMR problem. Instead, collective measures are needed by groups with greater autonomy than pig farmers [59], such as agro-industries, pharmaceuticals and animal health inspection bodies. Yet, the involvement of all stakeholders, including farmers, is essential to guarantee a sustainable transition to prudent AMU; as warned by von Keyserlingk and Hötzel [17], if changes in production systems are forced by external pressure, rather than by initiatives from the sector, decisions may not be fully linked to farmers’ concerns and priorities, which can generate economic risks for producers not prepared to respond.

Some farmers believed that changes in the rules for the use of antibiotics would come due to international demands, whereas national consumers and the risk of AMR, on the other hand, appeared as weak motivators for changes in behaviors regarding AMU. Farmers downplayed the role of Brazilian consumers as a driver of change, considering them not informed or interested in relation to the practices adopted on the farm. However, several studies have shown that lay citizens and consumers are increasingly concerned about issues related to livestock production, including the use of antibiotics [61,62,63] and some are aware of the relationship between AMR and the use of antibiotics in livestock production [64,65].

This study was restricted to the social context of one of the main pig-producing regions in Brazil. It is important to note that more quantitative and qualitative studies are needed to describe the situation regarding the use of antibiotics in a national context. In the meantime, the results of this study, even if localized, can provide us with a perspective of Brazilian farmers’ view on the problem of AMR and knowledge gaps that can be explored by other research focused on the prudent use of antibiotics.

## 5. Conclusions

Farmers are the direct guardians of pigs’ health and welfare; thus, they are essential for maintaining and complying with prudent AMU in the industry. Our qualitative study provides evidence of farmers’ unwillingness to adopt AMU practice changes, rooted in an unchallenged dependency on antibiotics. These farmers relied on antibiotics for disease prevention, whilst neglecting biosecurity and good animal welfare practices to reduce infection pressure and keep their pigs healthy. Equally, farmers reported mistrust, unpreparedness and misregulation from veterinary health services and the production chain. Altogether, this supports the AMU status quo, removing any pressure to change. Moreover, national public health concerns or consumers’ views did not compel them to change either. For this reason, we emphasize the importance of education and training of pig farmers and other rural workers regarding prudent AMU in pig farming and the risks of AMR. However, although transitioning to a more prudent AMU requires individual behavioral changes, we reinforce the idea that pig farmers are not sufficiently autonomous to determine substantial changes to reduce antibiotic use. Just as farmers indicated, we see external markets as positive catalysts for change. Yet, for this to work, national considerations and support structures have to be in place. If not, we forecast a forced loss of farmers’ diversity and increased stress in rural areas’ livelihood structures, which may increase the existent mistrust between farmers and regulatory institutions and national consumers.

## Figures and Tables

**Table 1:** Demographic characterization of the visited farms (*n* = 58).

Farm Type	Total *n* (%)
Farrow-to-finish	19 (33)
Breeding farms	26 (45)
Growing farms	4 (7)
Fattening farms	9 (15)
Herd size (number/herd)	
≤100 sows or finished pigs	7 (12)
101–500 sows or finished pigs	28 (48)
501–1000 sows or finished pigs	13 (22)
>1000 sows or finished pigs	10 (17)
Other farm activities	
Pig farming only	7 (12)
Dairy cattle	42 (72)
Aquaculture	10 (17)
Beef cattle	7 (12)
Other	1 (2)
Labor type	
Family and hired	28 (48)
Family	25 (43)
Hired	5 (9)

**Table 2:** Demographic data of the pig farmers (*n* = 58).

Gender	*n*	%
Male (M)	45	76
Female (F)	13	24
**Work experience**		
<5 years	3	5
6–10 years	5	9
11–15 years	11	19
16–20 years	5	9
>20 years	34	58
**Education**		
Elementary school	15 (10M, 5F)	26
High school	35 (30M, 5F)	60
Higher education	8 (5M, 3F)	14

**Table 3:** Biosecurity practices adopted by the farms visited.

Biosecurity Practices	Frequency *n* (%)			
	Never	Rarely	Sometimes	Always
Chlorine in drinking water	41 (71)	4 (6)	1 (2)	12 (21)
Rodent control	12 (21)	7 (12)	15 (26)	24 (41)
Visitor control	47 (81)	1 (2)	0	10 (17)
Vehicle control	48 (83)			10 (17)
	Never	<7 days	7–14 days	>14 days
Sanitary periods	17 (30)	24 (41)	14 (24)	3 (5)

**Table 4:** Infection prevention and control measures adopted on breeding farms.

Control of Infections in Sows *^1^	Total *n* (%)
Antibiotic in feed (antibiotic shock) every 6 months	19 (42)
Treatment in cases of present infection	7 (16)
Antibiotic in feed (antibiotic shock) every 4 months	6 (13)
Antibiotic in feed (antibiotic shock) every 3 months	4 (9)
Agro-industry control	4 (9)
Antibiotic in feed every 2 months	3 (7)
Antibiotic in feed for month	2 (4)
Control of infections in newborn piglets *^1,^*^2^	
Oral anticoccidial	26 (58)
Injectable antibiotic	20 (44)
Oral antibiotic	8 (18)
Oral prebiotic	2 (4)
Control of infections in weaning pigs *^3^	
Continued antibiotic use in feed	13 (41)
Antibiotic shock every feed change	12 (38)
Injectable antibiotic before weaning	4 (13)
Others	3 (9)

*^1^ % based on the number of farrow-to-finish and breeding farms (*n* = 45); *^2^ these items were cited more than once by the same participants, so the sum of citations is higher than the number of respondents; *^3^ % based on the number of farrow-to-finish, growing and fattening farms (*n* = 32).

**Table 5:** Most cited antibiotic groups by pig farmers.

Antibiotics in Feed	Antibiotic Groups	*n* (%)	Injectable Antibiotics *^1^	Antibiotic Groups	*n* (%)
	Aminopenicillins	24 (41)		Quinolones	36 (62)
	Tetracyclines	23 (40)		Aminopenicillins	34 (59)
	Amphenicols	19 (33)		Macrolides	19 (33)
	Pleuromutilins	13 (22)		Others	6 (10)
	Others	23 (40)			

*^1^ These items were cited more than once by the same participants, so the sum of citations is higher than the number of farmers. The percentages were calculated based on the number of participants (*n* = 58).

**Table 6:** Information on feed purchase and technical assistance.

**How do you get the feed?**	**Total *n* (%)**
Make on the farm	39 (67)
Is provided by the industry	17 (30)
Purchase	2 (3)
**Where do you buy veterinary antibiotics?**	
In agricultural stores	31 (53)
The agribusiness sells me the drugs	15 (26)
The agribusiness gives me the medicines	7 (12)
From feed supply seller	5 (9)
**Who do you receive technical assistance from?**	
Veterinary nutrition supply company	26 (45)
Integration or cooperative veterinarian	22 (38)
Private veterinarian	5 (9)
Veterinarian is part of the family	3 (5)
Agricultural technician at the agricultural store	2 (3)
**Frequency of visits by veterinarian**	
Weekly	20 (34)
Emergencies	19 (33)
Monthly	11 (19)
Biweekly	7 (12)
Daily	1 (2)

The percentages were calculated based on the number of participants (*n* = 58).

**Table 7:** Information on the most common diseases, means of diagnosis and treatment criteria described by the pig farmers.

Most Frequent Diseases *^1^	Total *n* (%)
Enteric diseases	40 (69)
Respiratory diseases	31 (53)
Encephalitis	10 (17)
Others	5 (9)
Do not know	4 (7)
**Diagnosis of diseases**	
Just from my experience	44 (76)
Veterinarian guidance	12 (21)
Agricultural technician guidance	2 (3)
**Antibiotic dose**	
Follow label directions	39 (67)
Follow the vet’s guidance	18 (31)
Follow the guidance of the agricultural store	1 (2)
Treatment time *^1^	
Veterinarian guidance	16 (28)
Follow label directions	16 (28)
1 to 3 days of treatment	15 (26)
Long-acting antibiotic (single dose)	11 (16)

*^1^ These items were cited more than once by the same participants, so the sum of citations is higher than the number of farmers. The percentages were calculated based on the number of participants (*n* = 58).

## Data Availability

Data may be available upon request by contacting the corresponding author.

## Supplementary Materials

The following are available online at https://www.mdpi.com/2079-6382/10/3/331/s1, Table S1: Script of the interviews with the demographic questions of the study; Table S2: Script of the interviews with the specific questions (biosecurity) of the study; Table S3: Script of the interviews with the specific questions (antibiotic) of the study.

## Appendix A

A. Scheme 30. a refers to the first excerpt from the interview with Farmer 30; F3b is the second excerpt quoted from the interview with Farmer 3.

F30a	I buy antibiotics in shops; I buy a bag [25 kg bag]. With the right dosage, I weigh it on a small scale, then mix it in the feed.
F57a	Yes, it is easy, you buy [antibiotics] in shops, like buying water at the bar.
F50a	The feed comes ready from the firm, the feed is already medicated, they put the antibiotics in it. They tell you when medicated feed will come, you have to go 28 to 30 days without carrying animals for slaughter, because you have to have a grace period, right?
F3a	I vaccinate [sic] with tulatromicine before weaning.
F1a	We control rats only when we see them. Visitors or vehicle control, we do not need that, not that many people enter here.
F2a	I don’t need chlorine because I have an artesian well. As for control of visitors, I have a book; sometimes visitors sign, other times they don’t.
F3b	You can’t raise pigs without antibiotics.
F19a	In order to not need antibiotics, we will need a stronger animals, more resistant to diseases.
F2b	You have to do the ’shocks’ every 6 months. The last time I did it was with florfenicol [amphenicols].
F57b	You have to do the preventions [sic], right? I use an antibiotic shocks three times a year on the entire herd, try to prevent [referring to diseases] so I don’t need to spend money all the time. I do it two or three times a year and I do it in all the herd.
F15a	You have to see the case, sometimes it is not worth treating [the sow]. It is better to eliminate her.
F17a	In the pre-initial diet, for post-weaning, it is amoxicillin [aminopenicillin] in the nursery, in the initial diet it is tiamulin [pleuromutiline], then one more shock at the start of the growing phase with tiamu-lin [pleuromutiline], and at the start of the finishing phase, florfenicol [amphenicols].
F19b	"There is always colistin [polymyxin], it goes in all diets, since the nursery phase, because you must have it. The others I interchange according to the market, the price of the antibiotic […] Colistin you must have. I can use an amoxicillin [aminopenicillin], a chlortetracycline [tetracicline], or maybe a tiamulin [pleuromutiline].
F41a	Piglets are vaccinated with tylosin [macrolides] when they are born […]. After 3 days, they get iron and anticoccidials.
F60a	I use injectable ceftiofur [cephalosporins] at birth. If I don’t, I have many piglets with arthritis during lactation.
F3c	Before weaning I vaccinate everyone with tulathromycin [macrolides].
F18a	In some farms they give the piglets tulathromycin at weaning [macrolides], it is a routine management.
F35a	Talking about the gilts… 3 doses are usually made, sometimes of tylosin [macrolides], amoxicillin or another injectable medicine, or in the diet with doxycycline [tetracyclines] or tiamulin [pleuromutilins].
F61a	Here we have very few diarrheas. Everything is controlled with the medicines they use in the sows’ feed. The main thing here is cleanliness inside the nursery, right?
F18b	In fact, the farm has its cycle of medication via feed, and we follow it up, by age. The cycle is continuous. There will always be some medicated animals inside the farm. Every day there is a medicated pig at some phase.
F30b	Diarrhea ends the piglet, sometimes from one day to the next it is already dead. We add just a ’little medicine’ [remedinho] to control it.
F51a	We do some ’little shocks’. We put medicated feed for 15 days, stop, leave a month open, add it for another 15 days…
F46a	We do a lot of prevention. Both sows and piglets are like children [meaning they get sick easily]. The ’shocks’ of the sows are done every 6 months, one shock during gestation and one during lactation. But during lactation it is a type of shock and during pregnancy it is another type. This is with the help of the vet, they do not usually give the same kind of shock because they say that it creates resistance.
F18c	A doxycycline bag [tetracycline] costs up to 6 thousand reais [equivalent to USD 1600].
F35b	You pay 250 reais [equivalent to USD 68] for 50 ml tulathromycin [quinolones].
F42a	The cost is high, it is expensive, but it is worth it because you will not lose the piglet.
F31a	Of course, if you have some type of disease, it is worth it. Because otherwise it ends up giving you a loss, so using the antibiotic will end up compensating.
F57c	Today the integrated famer receives the information faster […] Often the independent farmer, as he has to pay, take it out of his pocket to have a veterinarian, to see someone, which has a higher cost, then often he does not pay for it.
F61b	When you see that it is something you know, you go with confidence and apply the medicine. If it doesn’t work we call the vet to check it out.
F41b	For diarrhea, I ’vaccinate’ with tylosin [macrolide].
F60b	Some litters I do a single dose, others I give one the day, skip a day or two and repeat, often changing the medicine. Sometimes I do injectable oxytetracycline [tetracycline].
F35c	Another day a guy who sells feed ingredients told me to use tulathromycin [macrolide] at the same time as the iron. You take 3ml of tulathromycin and take 3ml of iron from the 100 ml, you mix it in the iron flask and then you do the iron together with the antibiotics, then it is cheap.
F18d	They get a antibiotic shock in the nursery, when they leave the nursery, and in growing pen. Thereafter, only if necessary, if there is an outbreak of influenza, or something that goes out of standard.
F59a	The last resort is to take samples to isolate the strain from the bacteria and send it out, to find out what it is.
F35d	They are similar [antibiotics for people and animals], so the Ministry of Agriculture wants us to stop using them. Usually children consume them; for example. Amoxicillin is one of the most used drugs in pigs and also one of the most used in children. That’s why they want us to avoid it, so as not to cause problems for children.
F1b	Resistance for me is using a medicine and in a little while it will have no effect. If I use it too long I have to change it, like amoxicillin and enrofloxacin every half year I change the medicine, if not, it no longer has an effect.
F14a	Antibiotics do not work; it is resistant on the farm; the disease is resistant to a certain antibiotic.
F58a	Low immunity in the animal; if there is a problem with it, no medication will work.
F37a	I don’t know for sure. I had a problem with the calves, we changed antibiotics and solved it. She gained immunity, I think that’s it.
F54a	Yes they worry, the less antibiotic you use, the better. Not only consumers, even us. Because if it shows up [referring to residues in the meat], I will not be able to sell anymore; today the piglets are all identified here. If there is a problem at the end of the chain, you will know that it is from my farm.
F20a	I think that a lot of people no longer eat pork because of that, they’re afraid of antibiotics, and then these swine flu things happen. All of this makes people suspicious of pork.
F25a	When we want to fatten up a pig to eat we do it separately, only with corn and without feed, without medicines, without anything.
F23a	…to catch a pig and kill it from the farm to eat it, I don’t have the courage.
F61c	Those who buy directly from us don’t need to worry. Because they come here looking only for those pigs that we separate for us to eat, without medicines.
F38a	If they cut the antibiotics it will be like this: it will decrease production, it will make the products more expensive, the poorer farmers are going to break […] I understand that today big companies want to end small ones.
F4a	I think it would cut production in half. To start the farmer needs to have money…
F2c	The point is that pig farming is very unstable. Most of the producers that we see are all drowning in debt… I think the laws are already well controlled, I don’t see this need…
F1c	Maybe I would have to adapt, do everything right from the beginning to the end. The way I work, it wouldn’t work. I will always need to use a little more antibiotics…
F58b	Facilities, increase the housing to have a greater sanitary period.
F9a	I think it wouldn’t work here [referring to Brazil] …
F32a	It may be, but in Brazil I think this will go a long way…
F42b	They can put the law in place, but it will be difficult to control it. … See colistin, the manufacture was prohibited, they simply went there, changed the label, instead of growth promoter they put antibiotics and released again…
F16a	I think it’s good, the problem is Brazil getting it. Today I don’t think so. We would have to change the production system a lot, but then with the costs, today we are already working a year in the red, the production costs are already high…
F33a	In the near future it will be restricted. I think it’s good, no more need to use so much antibiotics […] They have to try to do something so that they don’t need more antibiotics, a stronger animal, more resistant to diseases … also encourage people, other natural alternatives, who knows?
F22a	Animal welfare is also done to use fewer antibiotics. Like sows cannot stay in stalls, they have to stay more comfortable during gestation, they won’t get sick and you won’t need antibiotics.
