# Supplementary material for: Exploring Farmers’ Reasons for Antibiotic Use and Misuse in Pig Farms in Brazil
Source: Antibiotics (Basel). 2021 Mar 22;10(3):331. doi: 10.3390/antibiotics10030331 (PMC8004152; doi:10.3390/antibiotics10030331)
Supplement: Supplementary file 1 [file antibiotics-10-00331-s001.pdf]

Pigs can't be raised without antibiotics – Brazilian farmers' attitudes towards antibiotic use and antibiotic resistance: Supplementary Material

Rita Albernaz-Gonçalves[1,2,] Gabriela Olmos [3]and Maria José Hötzel [1*]

Supplementary Table S1: Script of the interviews with the demographic questions of the study

Demographic questions
Education
Elementary school
High school
Higher education
Sex
Male
Female
Time experience
Up to 5 years
Between 6 and 10 years
Between 11 and 15 years
Between 16 and 20 years
Type of farm production
Farrow-to-finish
Breeding farms
Growing farms
Fattening farms
Herd Size
Up to 100 finished pigs
Between 101 and 500 finished pigs
Between 501 and 1000 finished pigs
More than 1000 finished pigs
Who do you sell your animals to?
Independent
Cooperative
Integrator
Other:
Labour type
Family members only
Only hired people
Family and hired persons
Does the farm have other activities besides pig farming?

Table S2: Script of the interviews with the specific questions (biosecurity) of the study

Biosecurity issues	Frequency			
How often do you do these practices on your farm?	Always	Sometimes	Rarely	Never
Chlorine in drink water				
Rodent control				
Visitors control				
Vehicle control				
Sanitary period				
Never				
< 7 days				
7 -14 days				
> 14 days				

Table S3: Script of the interviews with the specific questions (antibiotic) of the study

**Technical assistance and disease diagnosis**
How do you identify which animals are sick?
How do you decide on the treatment of sick pigs?
Do you receive technical assistance on your farm?
How often do you receive technical assistance on your farm?
How do you get antibiotics and other veterinary drugs?
What is your opinion on the cost of antibiotics?
**Disease prevention and control**
What are the most common diseases on your farm?
Do you use medications on newborn piglets?
What medications do you use in newborn piglets?
How do you prevent genitourinary infections in sows?
How often do you supply antibiotics to the sows on your farm?
How do you prevent diseases in weaned piglets?
Could you name which antibiotics you use in the pigs' feed?
Could you name which injectable antibiotics you use in pigs?
Could you name which injectable antibiotics you use in pigs?
How do you define the dose and time of treatment with injectable antibiotics?
How do you define the dose and time of treatment with antibiotics in the feed?
In your experience, do antibiotics always work? And what do you do if they don't work?
**Knowledge and attitudes about the use of antibiotics and AMR**
Is there a difference between human and veterinary antibiotics?
In your opinion, are antibiotics used properly in pig farming?
In your opinion, what is bacterial resistance to antibiotics?
What factors induce bacterial resistance to antibiotics?
Have you heard about prudent antibiotic use policies?
Imagine a hypothetical scenario of antibiotic restriction in Brazil, in which the use of growth-promoting antibiotics were prohibited and the preventive use of antibiotics were restricted. What would be the viability of this scenario for Brazil and what would you consider necessary in order to reduce the use of antibiotics in pigs?
